# The Role of Distinct Cancer-Associated Fibroblast Subtypes in Prostate Cancer Immunotherapy

**DOI:** 10.7150/ijbs.132227

**Published:** 2026-08-12

**Authors:** Kankan He, Zhite Zhao, Jianhui Bai, Tong Lu, Yaohua Hu, Xinglin He, Fanchao Wei, Yixin Zhang, Yanan Gu, Zhaokun Shi, Xiangliang Meng, Fuli Wang, Changhong Shi, Weijun Qin, Keying Zhang, Qiaoli Xie, Lijun Yang

**Affiliations:** 1 Department of Urology, Xijing Hospital, Fourth Military Medical University, 710032, Xi'an, China.; 2 Joint Logistic Support Force, Hospital 981, 067000, Chengde, China.; 3 The Second Clinical Medical College of Shaanxi University of Chinese Medicine, 710000, Shaanxi, China.; 4 Division of Cancer Biology, Laboratory Animal Center, Fourth Military Medical University, 710032, Xi'an, Shaanxi, China.; 5 Department of Experimental Surgery, Tangdu Hospital, Fourth Military Medical University, 710038, Xi'an, China.

**Keywords:** prostate cancer, immunotherapy, cancer-associated fibroblasts, heterogeneity, tumor microenvironment

## Abstract

Immune checkpoint inhibitors (ICIs) have achieved breakthroughs in various solid tumors; however, their efficacy remains limited in prostate cancer (PCa), which is closely associated with its highly immunosuppressive tumor microenvironment (TME). As the most abundant stromal component in the TME, cancer-associated fibroblasts (CAFs) exhibit remarkable heterogeneity and functional plasticity, playing a central role in orchestrating an immunologically "cold" tumor phenotype and driving resistance to immunotherapy. We have moved beyond simple marker-based classification of cancer-associated fibroblast (CAF) subtypes — a framework still widely used in routine pathological assessment — and now define these populations by their functional states and molecular profiles, enabled by advances in single-cell sequencing, spatial multi-omics, and computational biology. Such functional divergence across stromal subsets allows individual CAF populations to engage in complex, multilayered crosstalk with different immune cell types, and together they build an immunosuppressive network that drives tumor immune evasion. This review systematically catalogs the identification methods and signature gene panels for distinct CAF subtypes in prostate cancer, with particular focus on the multilayered regulatory circuits through which these stromal cells shape the immune microenvironment. We also highlight emerging CAF-targeted strategies designed to reverse immunosuppressive states. This integrated perspective frames CAF heterogeneity in prostate cancer immune evasion, and establishes theoretical underpinnings and translational pathways for next-generation precision combination immunotherapies.

## Introduction

Global prostate cancer (PCa) incidence is projected to double worldwide, with disease-related mortality poised to rise by up to 85%, according to a major 2024 report in *The Lancet* 1. Therapeutic options remain extremely limited for advanced disease, most notably metastatic castration-resistant prostate cancer (mCRPC) 2, which is generally linked to dismal long-term prognosis; favorable clinical outcomes are reliably achieved in early-stage PCa via surgical resection and radiotherapy 3, 4. Such clinical disparities position the development of novel therapeutic strategies as a pressing unmet clinical priority.

Immunotherapeutic regimens built on ICIs and CAR-T-cell therapy have reshaped the treatment of immunogenic cancers, including melanoma 5, 6 and non-small cell lung cancer 7, 8. ICIs are known to elicit durable clinical remissions, and CAR-T-cell-based strategies are producing encouraging signals in select solid tumors, such as melanoma 9. In prostate cancer, however, these same immunotherapeutic approaches yield only marginal clinical benefit—an observation that sharply differentiates this malignancy from its immunogenic counterparts. The biological basis for this therapeutic disparity has been closely linked to the profoundly immunosuppressive tumor microenvironment (TME) of prostate cancer. The prostate TME is characterized by a dense infiltration of regulatory T cells (Tregs) 10, myeloid-derived suppressor cells (MDSCs) 11 , and M2-polarized macrophages 12. The cooperative activity of these immune cell populations establishes a niche that is permissive to tumor immune evasion 13. Restricted tumor immunogenicity is a hallmark of prostate cancer, driven largely by low mutational burden and subsequent impaired neoantigen generation 14. Phase III clinical data have validated such therapeutic constraints: the addition of the PD-1 inhibitor pembrolizumab to standard chemotherapy confers no overall survival benefit in patients with mCRPC 15. Even CDK12-altered mCRPC, a putatively immunogenic subgroup, yields a PSA50 response rate of only 9% to ipilimumab plus nivolumab 16. No clinically meaningful survival advantage is afforded by chemoimmunotherapy regimens over chemotherapy monotherapy in the mCRPC setting, as corroborated by a 2025 meta-analysis 17. The full therapeutic activity of ICIs and CAR-T-cell therapies depends on an immunologically permissive tumor microenvironment—a condition absent in prostate cancer, where the TME remains deeply immunosuppressive and immunologically "cold". Shifting this immunosuppressive, cold tumor microenvironment toward an immune-inflamed, hot phenotype has emerged as a key priority in translational prostate cancer research. Successful reprogramming of both stromal and immune compartments would unlock the full therapeutic potential of immune checkpoint inhibitors and CAR-T-cell therapies in this disease setting.

As documented in clinical cohort analyses, poor clinical outcomes in prostate cancer are tightly associated with high stromal content of cancer-associated fibroblasts (CAFs) 18, the dominant stromal cell population within the tumor microenvironment. Response rates to both immunotherapies and conventional chemotherapy are heavily shaped by CAF-driven immune modulation and extracellular matrix (ECM) remodeling 19. Effector function of T cells and natural killer (NK) cells is broadly suppressed by TGF-β, a core soluble effector secreted by stromal fibroblasts. Sustained inhibitory signaling pushes effector immune populations toward either exhaustion or a tolerogenic state, and such functional impairments, as corroborated by preclinical functional studies 20, abrogate their capacity to recognize and eliminate malignant cells. Chemokines such as CCL2 and CCL5 secreted by CAFs mediate recruitment of immunosuppressive cell subsets including tumor-associated macrophages (TAMs) and MDSCs, an effect independent of direct immune cell suppression that further amplifies the local immunosuppressive network 21. Dendritic cell (DC) maturation and antigen-presenting competency are impaired by CAF-derived signals, with consequent defects in the initiation of adaptive immune responses 22. These combined functional effects position CAFs as central drivers of the formation and sustained maintenance of an immunologically “cold” TME 23. Spatial distribution and functional states of discrete CAF subtypes can now be characterized with substantially enhanced precision, as multiplex immunofluorescence, in situ hybridization, organoid co-culture systems, and single-cell RNA sequencing (scRNA-seq) have gained broad implementation in tumor microenvironment investigations 24, 25. This review systematically explores both well-established and newly defined functional classification systems for CAF subtypes, drawing on data from preclinical models and clinical studies alike. We dissect in detail how individual CAF subpopulations modulate immune cell function and drive resistance to immune checkpoint blockade. Finally, we outline the translational promise of CAF-targeted treatments for prostate cancer. Computational tools designed to map cell-cell communication, when combined with these experimental approaches, have gradually revealed the complex regulatory networks that connect different CAF subsets to immune cell populations. This growing body of work has improved our understanding of how immunologically cold tumor microenvironments develop, and opens new paths to boosting the effectiveness of ICIs and CAR-T therapies.

## Conventional Classification of CAF Subtypes

Three major CAF subtypes are now broadly recognized across solid tumors: myofibroblastic CAFs (myCAFs), inflammatory CAFs (iCAFs), and antigen-presenting CAFs (apCAFs), with minor differences in naming and molecular markers seen across cancer types 26-28. Cancer-associated fibroblasts are defined first and foremost by their profound heterogeneity. These cells arise from a range of progenitor cell types, are found across nearly all solid malignancies, and become activated through complex signaling pathways 29, 30. Differences in cellular origin and activation state give rise to distinct phenotypic profiles, which in turn generate the functional diversity observed across CAF populations (Table 1). No single universal marker can reliably identify all CAFs in tumor samples. Researchers instead use a panel of canonical markers, including α-smooth muscle actin (α-SMA, *ACTA2*), fibroblast activation protein (FAP), platelet-derived growth factor receptors (PDGFRα/β), vimentin, and fibronectin 31, 32. These distinct fibroblast groups differ widely in their genomic and epigenetic profiles, spatial location within tumors, and functional outputs. Their combined activity shapes the full spectrum of tumor immune microenvironments, from immunologically cold to hot 33.

The first definitive delineation of two major CAF subtypes, myCAFs and iCAFs, in pancreatic cancer was reported by Öhlund et al. 34. TGF-β acts as the principal upstream driver of myofibroblastic CAF (myCAF) differentiation. Normal fibroblasts assume a high α-SMA myCAF phenotype after in vitro TGF-β exposure, and targeted blockade of this signaling axis, as demonstrated in functional studies 34, effectively abrogates such phenotypic conversion. The characteristic transcriptional signature of myCAFs features elevated expression of contractile protein-coding genes, including *Acta2* (α-SMA), *Tagln* (Transgelin), and *Myl9* 35. This stromal subset remains the most well-characterized and canonical CAF lineage reported across solid tumor research. In tumor tissue, myCAFs are largely restricted to collagen-dense regions in direct contact with tumor cells, a spatial distribution that aligns with their documented roles in ECM deposition and fibrotic remodeling 36, 37. The primary functional output of these cells centers on extracellular matrix synthesis and stromal restructuring, processes that drive progressive tissue fibrosis and desmoplastic matrix stiffening. As consistently reported across independent studies, the resulting fibrotic stromal compartment forms a biophysical barrier that impairs both chemotherapeutic drug delivery and intratumoral T-cell infiltration 38.

Inflammatory cancer-associated fibroblasts (iCAFs) drive chemotactic recruitment of Tregs and monocytes within the tumor stromal compartment via CXCL12-CXCR4 and CCL2-CCR2 signaling cascades 39. As characterized in prior transcriptional profiling work 40, this stromal subset exhibits robust induction of inflammatory mediators including IL6, CXCL12, CCL2, and COX-2, which form its core gene expression signature. PDGFRα, DPP4, and Ly6C constitute the canonical surface marker panel for identifying this fibroblast population. These cells accumulate preferentially in hypoxic and perivascular niches in tumor tissue, and maintain close spatial proximity to MDSCs and M2-polarized TAMs. iCAF-derived soluble factors — IL-6, PGE2, and HGF among them — inhibit dendritic cell (DC) maturation and diminish the cytolytic effector function of CD8⁺ T cells. An autocrine JAK/STAT3 feedback loop sustains the constitutive production of these stromal mediators 34.

Pronounced bidirectional fate interconversion is an inherent feature of iCAF and myCAF populations, neither of which exists as a static, terminally differentiated stromal phenotype. Distinct transcriptional reprogramming modules preserve the two discrete cellular states in pancreatic ductal adenocarcinoma (PDAC); α-SMA-dependent signaling stabilizes myCAF identity, whereas sustained IL-6 pathway activity maintains the characteristic iCAF phenotype 34. Colorectal cancer (CRC) studies have pinpointed Wnt signaling as the central regulatory determinant that governs fate transition between these two CAF subpopulations 41. An iCAF-to-myCAF phenotypic shift is elicited by anti-androgen therapy in prostate cancer, and this stromal reprogramming event is mechanistically mediated through the TGF-β-SOX4-SWI/SNF signaling axis 42. The biophysical ECM barrier deposited by myCAFs marks the boundary beyond which iCAFs are predominantly distributed. Efficient cytokine secretion from these spatially positioned stromal cells, as documented in prior studies 43, may promote recruitment of Tregs and other immunosuppressive cell populations. Pro-tumorigenic roles are proposed for this tissue arrangement, though the full functional relevance of iCAF-tumor cell spatial associations remains incompletely defined.

Two distinct apCAF subsets with differing cellular origins, tissue localization patterns, and functional properties can be identified across most solid tumor types. A pan-tissue fibroblast molecular atlas encompassing 15 normal tissues and solid tumor entities, assembled by Chen et al., classifies these two subpopulations as mesothelial-like and fibrocyte-associated subtypes 44. First described by Elyada et al. in 2019 via scRNA-seq of a murine pancreatic ductal adenocarcinoma (PDAC) model 45, the apCAF subset carries a defining phenotypic profile: surface expression of MHC class II molecules (e.g., *HLA-DRA, HLA-DPB1*) and CD74, coupled with a lack of canonical CD80/CD86 co-stimulatory molecules, placing it in the " non-professional " antigen-presenting fibroblast category. Follow-up studies have confirmed this stromal population across multiple tumor types, including gastric, lung, colorectal, and prostate carcinoma 46, 47. In gastric cancer tissue samples, apCAFs are selectively enriched at the periphery of tertiary lymphoid structures (TLS), directly adjacent to CD4⁺ T cells. This spatial localization pattern supports the utility of CD74high HLA-DRAhigh apCAFs as a pan-cancer predictive biomarker for immunotherapy response 46. In non-small cell lung cancer (NSCLC), patients achieving pathological complete response (pCR) or major pathological response (MPR) after neoadjuvant chemoimmunotherapy show markedly higher apCAF infiltration. These stromal cells act in concert with SELENOP⁺ macrophages to boost anti-tumor immune function 48.

## Identification and Immunomodulatory Functions of Novel CAF Subtypes in Prostate Cancer

Intratumoral clonal subpopulations and substantial interpatient molecular divergence, as validated by large-scale molecular profiling cohorts 49, constitute the defining clinical heterogeneity of prostate cancer. As corroborated by longitudinal clinical follow-up data 50, 51, three sequential phenotypic transitions delineate the stereotyped clinical trajectory of prostate cancer: initial presentation as hormone-sensitive prostate cancer (HSPC), progression to castration-resistant prostate cancer (CRPC) following sustained androgen deprivation therapy (ADT), and potential lineage transdifferentiation into highly aggressive neuroendocrine prostate cancer (NEPC). This inherent temporal dynamics of disease progression has driven a shift in CAF research approaches within human solid tumor cohorts. Static cross-sectional case-control designs are no longer the dominant analytical framework. Modern study designs instead center on longitudinal disease evolution, and incorporate comparative analyses of primary and metastatic lesions, pre- and post-treatment states, and distinct clinical stages.

Researchers now deploy a suite of high-throughput methodologies to delineate CAF spatial distribution patterns, phenotypic profiles, and functional states 23-25, 31, each carrying distinct analytical strengths and inherent technical constraints. Single-cell RNA sequencing (scRNA-seq) enables unbiased subtyping of CAF populations and definition of their molecular signatures 23, 31 but by the nature of its dissociative workflow, it loses native spatial localization context. Spatial transcriptomic platforms such as Xenium preserve intact tissue spatial coordinates and support in situ profiling of CAF—tumor—immune crosstalk 29, 31, though broader application is constrained by relatively low throughput and high assay costs. At the protein level, co-detection by indexing (CODEX) and multiplex immunofluorescence (mIF) achieve single-cell resolution visualization of CAF markers and immune cells in tissue sections 24, 25, with the inherent trade-off of a limited panel of detectable targets. Spatial mapping of CAF functional states at single-cell resolution can be achieved with imaging mass cytometry (IMC), a platform that couples cellular imaging with detection of upwards of 40 protein markers to support deconvolution of stromal landscapes, though it entails a comparatively laborious analytical workflow. Complementary technical merits across transcriptomic and proteomic modalities underpin the prevailing investigative framework, which incorporates a suite of tools spanning scRNA-seq, spatial transcriptomics, and high-dimensional spatial proteomic platforms such as IMC and CODEX. Precise dissection of CAF subtype spatial heterogeneity and functional states in prostate cancer is broadly recognized as optimally addressed by such combinatorial multi-omic strategies 29, 31.

Two complementary analytical dimensions underpin the systematic delineation of prostate cancer-associated fibroblast subtypes presented herein: functional stratification and gene signature-guided subtyping. Phenotypic state shifts of these stromal subsets across temporal disease progression are detailed, and their clinical applicability for prognostic stratification and immunotherapeutic response forecasting is also covered.

### CAF Subtypes Classified by Functional Characteristics in Prostate Cancer

**Figure 1:**
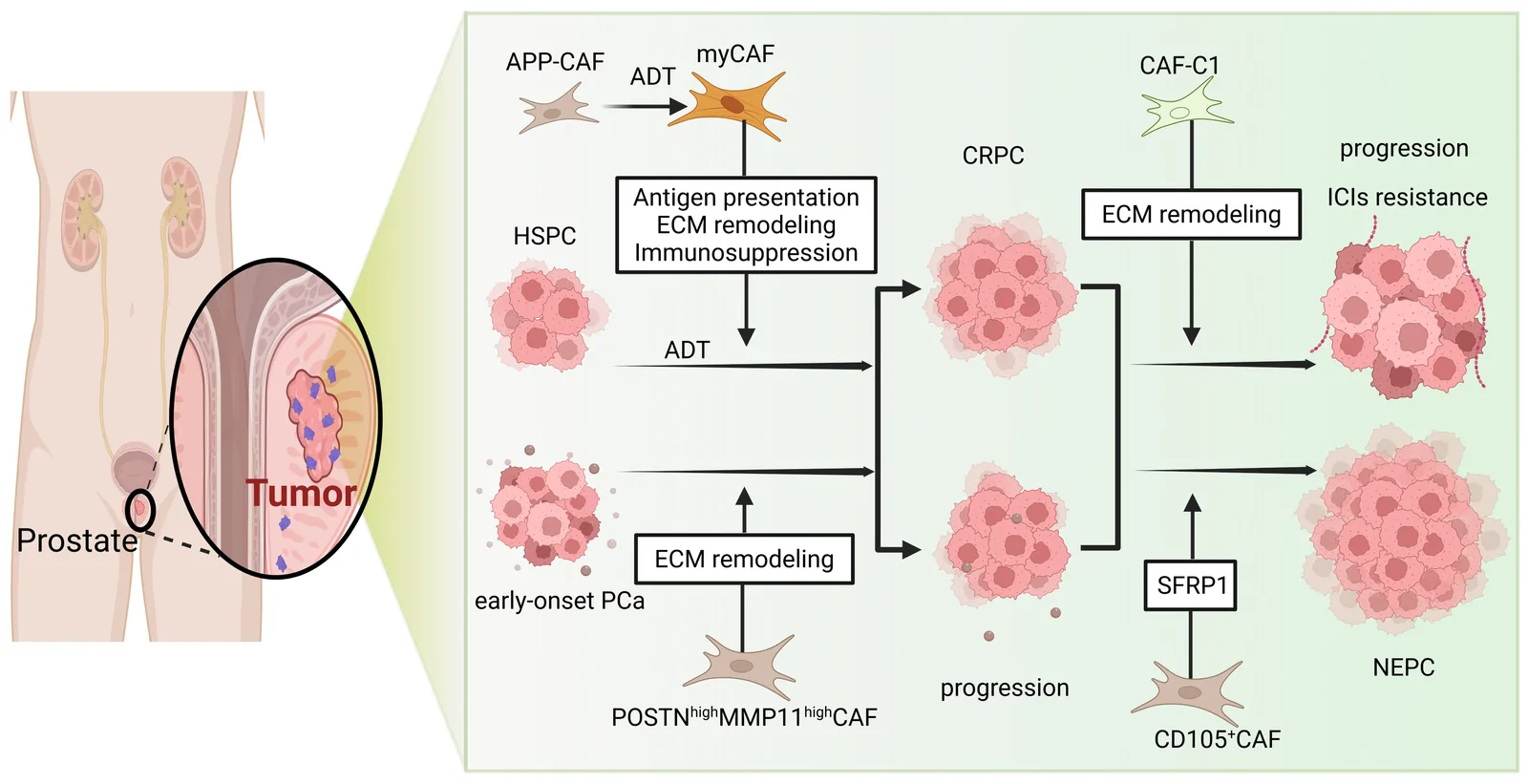
Subtype conversion of CAFs during the progression of prostate cancer.

The tumor immune microenvironment across prostate cancer progression is shaped by dynamic, functionally distinct CAF subtype transitions (Figure 1). Early-stage prostate cancer such as primary HSPC harbors dominant immunoregulatory/inflammatory ECM-CAFs, a subset that phenotypically aligns with the iCAF subtype. These CAFs exhibit high expression of inflammatory factors and antigen-presentation-related genes, endowing them with dual functions: recruiting immune cells and potentially activating T cells 52. However, upon ADT and disease advancement, their antigen-presenting capacity is often diminished, and their function shifts toward profound immunosuppression—for instance, by secreting Gas6 to activate macrophage efferocytosis 53. Concurrently, ADT releases TGF-β signaling, driving a SOX4-SWI/SNF-dependent conversion of inflammatory CAFs into SPP1⁺ myofibroblastic CAFs (myCAFs) 42. This suggests that ADT monotherapy may inadvertently create an unfavorable milieu for subsequent immunotherapy.

Simultaneously, ECM/myofibroblastic CAFs (myCAFs/ECM-CAFs) in primary tumors physically impede immune cell infiltration through excessive deposition and remodeling of the ECM 54. In more aggressive early-onset prostate cancer, a CAF subpopulation highly expressing POSTN and MMP11 exacerbates this process 55. Accordingly, targeting such pro-fibrotic, pro-invasive CAF subsets represents a high-priority strategy for early-onset patients to breach the dense ECM barrier.

As disease advances to CRPC, CAFs-C1 emerges as the predominant subtype, marked by elevated *FN1* and *FAP* expression; this subset functionally corresponds to myCAFs overexpressing both markers. Beyond directly driving disease progression, this robust ECM-remodeling activity correlates with ICI resistance 56. For patients with CRPC or advanced disease, therapeutic efforts should center on immunosuppressive CAF subsets enriched in CRPC specimens or their secreted factors; such targeting underpins effective combination immunotherapy.

Progression to highly aggressive NEPC coincides with expansion of a CD105⁺ CAF subset that encircles neuroendocrine-differentiated epithelial cells and drives paracrine neuroendocrine differentiation of prostate cancer cells via SFRP1 secretion 57. Therapeutic strategies targeting this CAF subset may effectively halt NEPC progression.

Elevated intratumoral infiltration of immunosuppressive cell populations such as Tregs and MDSCs, coupled with blunted immunotherapeutic efficacy in high-risk patient cohorts, correlates robustly with high-lactate-metabolism-associated cancer-associated fibroblasts (HighLM-CAFs) 58, 59. An immunosuppressive tumor stromal milieu is orchestrated by this CAF subset via altered lactate metabolism. Functional pleiotropy is prevalent across distinct CAF subtypes, and many subsets simultaneously exhibit stromal remodeling and immunosuppressive capacities. Such dual phenotypes are observed in CAFs positive for mesenchymal stem cell markers (CD90⁺CD105⁺). These cells mediate pro-fibrotic effects and myeloid suppressor cell recruitment, and intratumoral TAM and MDSC infiltration is driven by secreted soluble mediators with CCL2 as a key component 57.

Robust activation of the TGF-β signaling cascade during cancer-associated fibroblast phenotypic reprogramming has been validated in human prostate cancer specimens via single-cell sequencing by Qiu and colleagues; this pathway functions as a principal driver of such CAF phenotypic remodeling. TGF-β1 stimulation augments fibroblast proliferative potential and migratory capacity in vitro. WPMY-1 cells exposed to TGF-β1 exhibit concurrent activation of downstream TGF-β and MYC pathways, as well as marked suppression of interferon responses and inflammatory cascades 60. Such systematic transitions in CAF subtypes, which shift from a potentially immune-activating profile toward strongly immunosuppressive and pro-fibrotic phenotypes, align with prostate cancer progression from early-stage disease to advanced states, most notably castration-resistant prostate cancer (CRPC). Targeted inhibition of TGF-β signaling thus holds promise for reversing the immunosuppressive phenotype mediated by CRPC-associated CAFs, providing a novel interventional direction to remodel the tumor immune microenvironment and boost immunotherapy responses. Tumor invasion, metastatic spread, and therapeutic resistance are driven by a progressively deteriorating immunosuppressive microenvironment shaped by the aggregate of these cellular perturbations.

### CAF Subtypes Defined by Characteristic Gene Signatures and Associated Prognostic Models

Bulk transcriptomic profiles from large primary tumor cohorts, exemplified by the TCGA-PRAD dataset, and single-cell transcriptomic datasets spanning diverse stages of disease progression underpin the majority of relevant investigations. CAF subtype classifiers and gene signatures with prognostic and therapeutic predictive utility are devised by multiple research groups via the integration of key marker genes. As one example, single-cell analysis led Pan et al. to identify two principal CAF subtypes: αSMA⁺CAV1⁺ CAFs-C0 and FN1⁺FAP⁺ CAFs-C1. The CAFs-C1 subtype was significantly enriched in CRPC samples, and its high expression score correlated with poor patient prognosis and resistance to ICIs 56. Similarly, Wang et al., using non-negative matrix factorization clustering, identified a CTSK⁺MRC2⁺ CAFs-C1 subtype in primary tumors associated with antigen presentation function 52. Furthermore, a pan-cancer study systematically categorized CAFs into six major subtypes and confirmed their presence in primary prostate tumors (TCGA cohort); integrating these subtype signatures into a total CAF (tCAF) score revealed that a high tCAF score correlated with specific immune-activated microenvironment phenotypes 61.

Regarding prognostic model construction, several studies have developed clinically applicable risk-scoring tools based on CAF-related transcriptional signatures. For example, a four-gene model (*THBS2*, *DPT*, *COL5A1*, *MARCKS*) derived from antigen-presenting-related CAF subtypes stably predicted biochemical recurrence risk in the TCGA cohort and multiple independent GEO validation sets 52. A ten-gene risk signature constructed by Wen et al. was also validated as an independent prognostic factor for poor outcomes in primary cancer, with the high-risk group significantly associated with an immunosuppressive TME and poorer ICI treatment response 62. Another study calculated the expression ratio of "pro-tumor" versus "anti-tumor" CAFs gene sets to build a CAFs functional index, finding that low expression of this index in primary cancer samples predicted worse clinical outcomes 63. At the epigenetic regulation level, Xu et al. constructed a 9-miRNA prognostic model based on single-cell data from 34 primary prostate cancer samples and functionally validated the pro-tumor role of *hsa-miR-106b-5p* in CAFs 64. The construction of such prognostic models enables the early identification of patients at high risk of recurrence, thereby providing crucial guidance for adjuvant treatment decisions.

It is important to note that multiple nomenclature systems currently exist for CAFs in prostate cancer, including classical functional classification (myCAFs, iCAFs, apCAFs) 26-28, disease stage-specific classification (CAFs-C0, CAFs-C1), metabolism-based classification (HighLM-CAFs, FerroCAFs) 58, and molecular marker-based classification (CD105⁺ CAFs, ADAM12⁺ CAFs) 56, 65, among others. Some functional categories overlap, which can muddy the interpretation across studies. Four spatially defined CAF subsets (s1-s4) exhibit robust conservation across diverse tumor entities and spatial omics profiling platforms 31. These findings were reported by Liu and colleagues in a recent *Cancer Cell* publication, where the team performed single-cell spatial multi-omics assays on ten human cancer types. Such a spatially anchored categorization approach advances progress toward a unified pan-tumor CAF framework and may resolve inconsistencies that arise from competing nomenclature systems.

### Mechanisms of CAF-Mediated Regulation of the Prostate Cancer Immune Microenvironment

An immunosuppressive stromal niche is formed through the concerted action of heterogeneous cancer-associated fibroblast (CAF) subsets via distinct molecular programs, and this specialized microenvironment drives tumor immune escape and the development of therapeutic resistance. The tumor immune microenvironment within solid tumor lesions is extensively remodeled by these stromal cells via a broad spectrum of mechanistic pathways, which include cytokine secretion, physical stromal barrier construction, metabolic remodeling, and exosome-mediated intercellular communication (Table 2). Tumor progression and the modulation of host immune responses are under the pivotal regulatory control of CAFs.

### CAFs Directly Impair CD8⁺ T Cell Infiltration and Function

Effective anti-tumor immunity hinges on sufficient intratumoral infiltration of cytotoxic CD8⁺ T cells and intact effector function. CAFs promote immune evasion and tumor progression through multiple mechanisms, including impeding CD8⁺ T cell infiltration via ECM components, mediating intercellular communication through exosomes, and upregulating PD-1 expression via secreted chemokines. Research by Song et al. identified two functionally opposed CAF subtypes: ECM-associated CAFs (ECM-CAFs), induced by TGF-β and characterized by a YAP1-centric transcriptional program with high expression of CD248/COL1A1, which promote collagen deposition and accelerate tumor progression; and lymphocyte-associated CAFs (Lym-CAFs), induced by TNF-α/IFN-γ (primarily secreted by lymphocytes) and driven by NF-κB p65, with high expression of TSG6/CXCL9/10/11, which recruit and activate CD8⁺ T cells to exert anti-tumor effects.

Mechanistically, YAP1 binds directly to IKKα, inhibiting its phosphorylation and thereby blocking the nuclear translocation and activation of NF-κB p65, locking CAFs into the pro-tumorigenic ECM-CAF phenotype. Inhibiting or conditionally knocking out YAP1 in CAFs relieves this suppression of the NF-κB pathway, driving the conversion from ECM-CAFs to the immunostimulatory Lym-CAF phenotype. Specific ablation of YAP1 in prostate CAFs effectively reduces collagen deposition and increases CD8⁺ T cell infiltration and activation 66. Similarly, monoamine oxidase A (MAOA), an emerging therapeutic target in prostate cancer, inhibits the differentiation of myCAFs into iCAFs. Attenuated tumor growth, elevated tumor-infiltrating CD8⁺ T cell proportions, and increased IFN-γ levels in tumor tissue lysates stem from MAOA modulation in cancer-associated fibroblasts 67. Augmented WNT5A secretion mediates these phenotypic alterations. Either MAOA downregulation in CAFs or pharmacological inhibition triggers this secretory phenotype, which engages the Ca²⁺-NFATC1 signaling cascade to drive downstream functional effects. Suppression of collagen deposition and skewed CAF differentiation toward an immune-potentiating phenotype efficiently reshape the tumor immune microenvironment and bolster CD8⁺ T cell infiltration and anti-tumor cytotoxic function.

Long-range modulation of CD8⁺ T cell responses within the tumor microenvironment is mediated by CAF-secreted exosomes 68, which harbor bioactive molecules including microRNAs, proteins, and lipids 69, 70. Proliferation, migration, invasion, and viability of prostate cancer cells are robustly promoted by CAF-derived exosomes, while exosomes from normal fibroblasts (NFs) exert minimal effects in comparative assays 71. CD8⁺ T cells show blunted proliferation and elevated apoptosis upon exposure to these vesicles, which supports their role in tumor immune evasion. Subsequent assays detect abundant PIK3AP1 expression in CAFs, their secreted exosomes, and prostate cancer cells. CAF-derived exosomes exert dual pro-tumorigenic effects. They directly drive malignant progression and impair CD8⁺ T cell viability via growth arrest and apoptosis induction, effects that enable tumor immune evasion 72.

Chemokine secretion provides an exosome-independent mechanism for CAFs to modulate CD8⁺ T cell function 73, 74. Isolating and performing RNA sequencing on CAFs from benign and matched malignant prostate cancer tissues revealed significant upregulation of CCL5 in malignant CAFs. Analysis of TCGA-PRAD data further showed a positive correlation between CCL5 and PD-L1 expression. Mechanistic studies demonstrated that CCL5 binding to its receptor CCR5 activates the AKT signaling pathway, leading to upregulated PD-L1 transcription and protein expression. Blocking this axis with the CCR5 antagonist maraviroc or the AKT inhibitor MK-2206 confirmed that CCL5 enhances PD-L1 expression via the CCL5-CCR5-AKT pathway to promote tumor immune evasion 75. Furthermore, in the TRAMP transgenic mouse model of prostate cancer, specific upregulation of stromal FOXF2 significantly delays tumor progression and distant metastasis. scRNA-seq analysis indicated an increased proportion and enhanced function of intratumoral CD8⁺ T cells, alongside a shift in CAF phenotype from inflammatory to myofibroblastic with reduced immunosuppressive activity. Mechanistically, FOXF2 directly binds to and inhibits the promoter activity of the chemokine *CXCL5*, while indirectly suppressing the NF-κB and STAT3 signaling pathways, thereby significantly reducing CXCL5 expression 76. Thus, targeting chemokines and their associated signaling pathways can also effectively disrupt CAF-mediated immunosuppression.

### CAFs Establish an Immunosuppressive Barrier by Regulating Macrophage Polarization

Spatial colocalization of cancer-associated fibroblasts and M2-like macrophages is consistently observed in prostate cancer tissue specimens across multiple disease stages, and higher abundances of both cell populations typically correlate with more advanced tumor grade and elevated metastatic risk. Cytokines secreted by cancer-associated fibroblasts represent a major driver of this M2-skewed polarization shift. Macrophages display two primary polarized phenotypes: anti-tumor M1 and pro-tumor immunosuppressive M2. Distinct extracellular signals trigger these functional states, which reflect the marked plasticity inherent to these key innate immune cells 77, 78. Most macrophages infiltrating the tumor microenvironment adopt an M2-like tumor-associated macrophage phenotype, and these cells sustain the immunosuppressive properties of the local stromal compartment 79, 80. Monocyte commitment to an M2 polarized phenotype occurs during in vitro co-culture with cancer-associated fibroblasts, and this lineage transition is mediated by secreted factors including IL-6, M-CSF, and TGF-β. Reciprocal stromal signaling from these polarized macrophages acts on CAFs via CCL18, IL-10, and additional soluble mediators, and establishes a self-amplifying feedback circuit within the stromal niche. Core cancer cell phenotypes, including migratory capacity, invasive potential, epithelial-mesenchymal transition (EMT), and stem-like properties, are all augmented by the synergistic crosstalk of these two stromal populations, and this coordinated functional effect accelerates overall tumor progression 81. Zhang et al. found that high expression of the estrogen receptor GPR30 in prostate cancer stromal fibroblasts upregulates the secretion of CXCL12 and IL-6, recruiting macrophages and inducing their M2 polarization to enhance tumor invasion. Knockdown of *GPR30* blocks this axis, reducing macrophage infiltration and M2 polarization and diminishing the pro-tumorigenic capacity of CAFs 82. In neuroendocrine prostate cancer (NEPC), the IL-8/CXCR2 signaling axis promotes CD47 expression and M2-like TAM polarization through lipid metabolic reprogramming, driving immune evasion in NEPC. Preclinical studies show that targeting CXCR2 restores TAM phagocytic activity and significantly reduces tumor growth 83.

Furthermore, during the early stages of TME formation, TGF-β signaling can induce a subpopulation of stromal cells expressing ADAM12 65. These cells, by secreting high levels of the efferocytosis bridging molecule Gas6, engage the AXL receptor on macrophages, significantly enhancing macrophage clearance of apoptotic tumor cells. This process activates the STAT1/3-SOCS signaling axis, driving macrophage functional reprogramming: suppressing their pro-inflammatory M1 program while inducing the secretion of immunosuppressive M2-like factors such as TGF-β and IL-10, thereby establishing a "phagocytosis leads to suppression" vicious cycle 53. Subsequently, the reprogrammed macrophages downregulate secretion of the CXCL9/10 chemokines and upregulate the loss of ICAM1 on vascular endothelium, dually blocking CD8⁺ T cell trans-endothelial migration and adhesion 84. This mechanism maintains local tumor hypoxia and acidosis, which in turn persistently suppresses T-cell glycolytic metabolism and effector function. Notably, specific depletion of ADAM12⁺ stromal cells reverse M2 macrophage polarization and restores T cell infiltration and IFN-γ production. Preclinical studies confirm that this immune evasion mechanism is highly conserved in human pancreatic, prostate, and melanoma cancers and is closely associated with resistance to ICIs, making targeting this cell population or its downstream signals a promising new strategy to overcome immune evasion 53.

### CAFs Regulate Other Lymphocyte Subsets

The immunomodulatory role of CAFs is broad, extending beyond macrophage polarization and CD8⁺ T cells to directly interfere with the function and differentiation of other key immune cells 85. For instance, lactate produced by CAFs via high glycolysis, on one hand, degrades the transcription factor T-bet in a SIRT1-mediated manner, thereby inhibiting the differentiation of anti-tumor Th1 cells. On the other hand, it activates the NF-κB pathway, promoting the generation of immunosuppressive Tregs, further enhancing the immunosuppressive state within the TME. CAFs also influence the recruitment of MDSCs 86. Downregulation or loss of the transcription factor FOXF2 in stromal cells relieves its inhibition on the chemokine CXCL5. Elevated CXCL5 expression subsequently recruits large numbers of MDSCs into the TME, which then suppress T cell activity 87. Researchers have identified a subclass of iron-laden tumor-associated fibroblasts (FerroCAFs) in prostate cancer. These FerroCAFs upregulate *Hmox1*, leading to iron accumulation and activation of the iron-dependent histone lysine demethylase Kdm6b, which induces the secretion of myeloid cell-associated proteins including CCL2, CSF1, and CXCL1. This, in turn, recruits immunosuppressive myeloid cells, shaping an immunosuppressive TME 87. Additionally, studies indicate that CAF cells with higher estrogen receptor α expression (CAFs.ERα(+)) inhibit prostate cancer invasion by suppressing the secretion of chemokines CCL5, IL6, and macrophage infiltration within the TME 88.

### CAF Subtype Interactions in Shaping the Prostate Cancer Immune Microenvironment

Complex cooperative and counterregulatory functional networks emerge from phenotypically heterogeneous CAF subsets in prostate cancer, as individual stromal populations do not execute their biological functions in complete isolation.

Notably, specific CAF subpopulations play critical roles in this process. For instance, multiple studies have demonstrated that myCAFs/ECM-CAFs, through excessive deposition and remodeling of the ECM, construct a dense stromal physical barrier that impedes immune cell infiltration 56, 89. Concurrently, iCAFs/APP-CAFs establish an immunosuppressive network by secreting cytokines such as IL-6, CCL2, CXCL12, and TGF-β1 34. These two subpopulations act synergistically to form a dual "physical barrier plus immunosuppression" defense that collectively sustains the immunologically "cold" TME. In castration-resistant prostate cancer, αSMA⁺CAV1⁺ CAFs-C0 are associated with stromal remodeling, whereas CAFs-C1 are closely linked to immunosuppression; together, they further reinforce stromal remodeling and amplify immunosuppressive signaling, thereby promoting immunotherapy resistance 56.

Conversely, in early-stage tumors, apCAFs are relatively enriched and exhibit antigen-presenting and immune-activating functions that promote T cell activation and anti-tumor immunity, thereby antagonizing the immunosuppressive functions of myCAFs and iCAFs 45, 52. Furthermore, by integrating four prostate cancer scRNA-seq datasets, researchers have identified two functionally opposing CAF subtypes: ECM-associated CAFs (ECM-CAFs), which promote collagen deposition and tumor progression, and lymphocyte-associated CAFs (Lym-CAFs), which display an anti-tumor phenotype and induce CD8⁺ T cell infiltration and activation 66.

**Figure 2:**
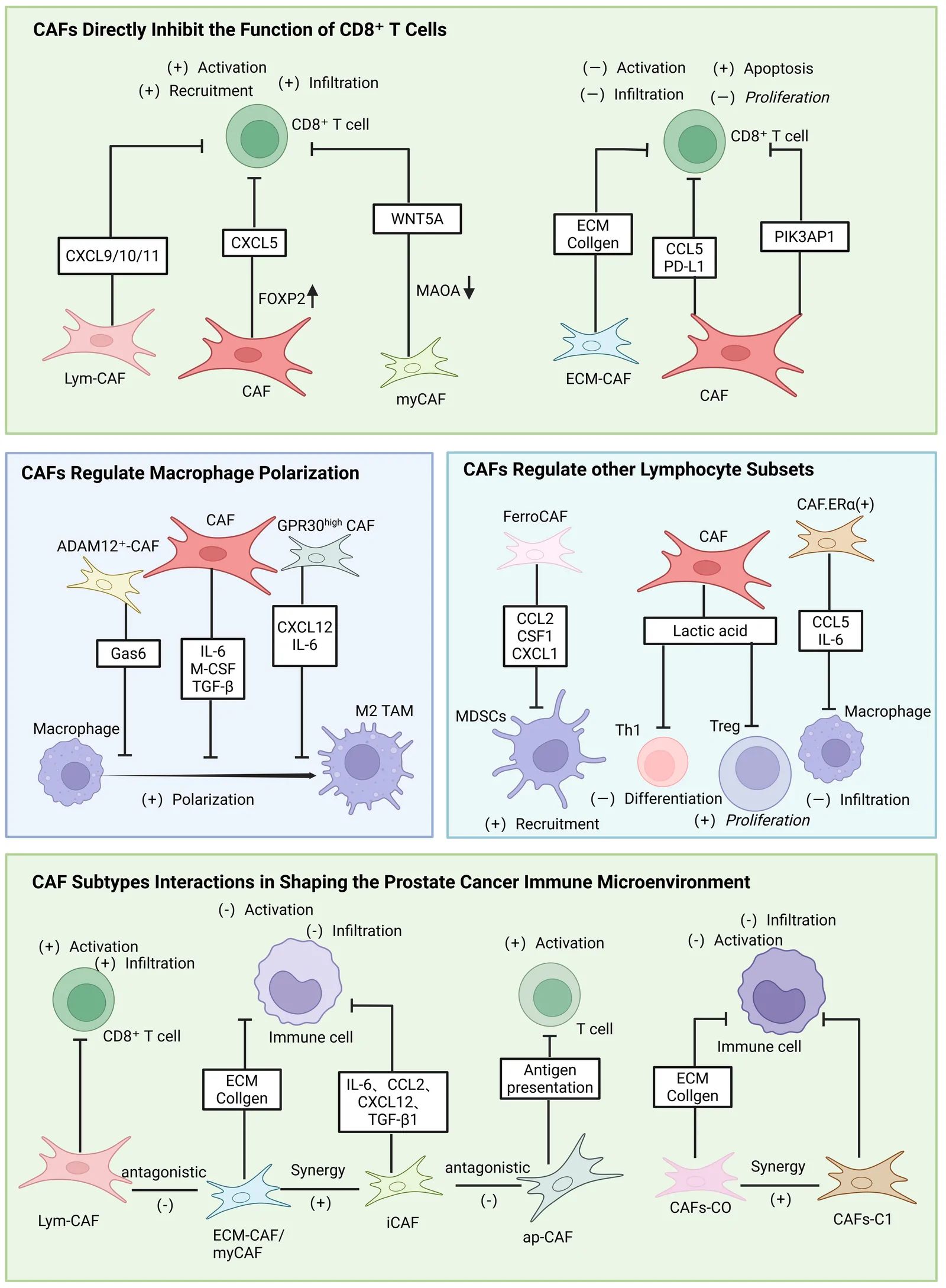
** Mechanisms of CAF-mediated regulation of the prostate cancer immune microenvironment.** Specifically, the interplay among distinct CAF subtypes collectively shapes the tumor immune microenvironment. CAFs directly impair CD8⁺ T cell infiltration and function, including suppressing T cell activation and proliferation, inducing T cell exhaustion and apoptosis, impeding T cell infiltration, and influencing T cell differentiation. CAF-mediated macrophage polarization drives the formation of an immunosuppressive barrier, with further regulatory effects on other immune cell subsets including Th1 cells, Tregs, MDSCs, and macrophages.

In conclusion, CAFs are no longer viewed as passive supportive elements of the tumor stroma but are recognized as active, core regulators that shape the immunosuppressive TME. Distinct CAF subtypes engage in complex interactions with each other and with diverse immune cell populations—including macrophages, T cells, and MDSCs—thereby constructing a multi-layered immune evasion network (Figure 2). Consequently, targeted intervention strategies against CAFs have emerged as novel therapeutic targets.

## Potential Therapeutic Strategies Targeting CAFs

CAFs drive tumor progression in multiple ways, which makes them attractive targets for therapy. In prostate cancer, most strategies aimed at CAFs seek to eliminate cells bearing specific surface markers or to cut off key signaling routes. At the same time, newer drug designs are showing considerable potential — oncolytic viruses and antibody-drug conjugates, for example, can elicit meaningful antitumor immunity. Furthermore, natural compounds have demonstrated important roles in modulating CAF functions (Table 3).

### Targeting CAF-Specific Biomarkers and Signaling Pathways

Fibroblast activation protein (FAP), a specific marker of CAFs, is highly expressed in nearly all epithelial cancers with restricted expression in normal tissues, making it an attractive therapeutic target 90, 91. A nanoparticle system delivering FAP antibodies can inhibit CAF activity by downregulating CXCL12 expression, thereby modulating the PCa TME 92. Among distinct CAF subtypes in prostate cancer, FAP-expressing CAFs are highly pro-tumorigenic. Researchers have developed a FAP-targeting antibody-drug conjugate (FAP-ADC, huB12-MMAE) by coupling the anti-FAP antibody huB12 with the cytotoxic payload monomethyl auristatin E. This agent induces necroptosis in CAFs by releasing its payload upon internalization, disrupting CAF microtubule structure 93. High levels of EFNB1 and EFNB3 in benign human prostate stromal cell lines enhance the tumorigenicity of PCa cells and activate Src family kinases (SFKs) in prostate fibroblasts 94. SFKs play a pivotal driving role in prostate cancer progression, and multiple therapeutic strategies targeting SFK intervention have been developed. Researchers have employed the small-molecule SFK inhibitor dasatinib to effectively suppress the expression of activated SFKs, thereby inhibiting tumor growth and lymph node metastasis development in both androgen-sensitive and androgen-resistant tumors 95. *In vitro* experiments demonstrated that the SFK inhibitor AZD0530 reduced the expression of the CAF marker α-SMA and the ECM protein tenascin-C (TNC) 94. In clinical trials, however, the SFK inhibitor AZD0530 exhibited limited efficacy as monotherapy in patients with castration-resistant prostate cancer 96. MPSSS, a natural polysaccharide extracted from *Lentinula edodes* (shiitake mushroom), disrupts CAF-mediated T cell suppression by activating the TLR4-NF-κB signaling pathway 97. Meanwhile, the Chinese herbal active component cinnamaldehyde, by inhibiting activation of the TLR4/NF-κB signaling axis, significantly reversed CAF-mediated suppression of T cells and restored their anti-tumor function without causing substantial cytotoxicity 98. Notably, silibinin reduces the expression of certain CAF biomarkers and TGF-β2 in prostate cancer, thereby inhibiting the conversion of normal fibroblasts to CAFs, highlighting the therapeutic potential of such natural substances 99. In prostate cancer, CAFs promote tumor growth and chemoresistance by secreting angiopoietin-like protein 4 (ANGPTL4), which binds to IQ motif-containing GTPase-activating protein 1 (IQGAP1) on cancer cell membranes, activating the Raf-MEK-ERK-PGC1α axis to promote mitochondrial biogenesis and OXPHOS metabolism. Quercetin-3-O-β-D-glucose-7-O-β-D-gentiobioside (QGGP), a specific inhibitor of IQGAP1, combined with docetaxel treatment, can reverse CAF effects and improve PCa chemosensitivity 19. Shifts in CAF functional phenotype within the prostate tumor microenvironment that confer enhanced tumor sensitivity to immune checkpoint blockade (ICB) can be triggered by the transcription factors YAP1 and FOXF2 alongside the mitochondrial enzyme MAOA 66, 67, 76. Strong associations between PPAR, GnRH, and mTOR signaling cascades and prostate cancer pathogenesis have been identified through genomic variant profiling in patient-derived samples by Zhai and co-workers, and these findings support these pathways as candidate actionable therapeutic targets 100. Pioglitazone, a PPAR agonist, has been assessed in preclinical models and early-phase clinical studies. Prostate cancer cell proliferation is suppressed by this agent, which also induces metabolic alterations and epithelial phenotypic changes 101, 102. Reduced luteinizing hormone and follicle-stimulating hormone secretion from pituitary gonadotrophs, and a subsequent decline in testosterone production, are induced by GnRH antagonist binding to pituitary GnRH receptors. Clinical approval for advanced prostate cancer has been granted to the GnRH antagonist relugolix 103, and a phase II trial of teverelix DP has concluded 104. Combined use of the pan-PI3K/mTOR inhibitor gedatolisib and darolutamide in mCRPC patient cohorts is being evaluated in an ongoing phase I/II trial.

### Targeting CAF-Induced Castration Resistance

Acquired therapeutic resistance invariably emerges with extended treatment durations, although androgen deprivation therapy (ADT) serves as the central therapeutic mainstay for prostate cancer clinical care. Multiple mechanistic pathways underlying this resistance phenotype are mediated by cancer-associated fibroblasts (CAFs), which have been established as pivotal stromal regulators in this pathological process 42, 105. A more aggressive tumor phenotype is induced by the co-culture of castration-resistant prostate cancer stem cells with castration-resistant prostate cancer-associated fibroblasts (CRPC-CAFs), as documented by Adisetiyo et al. Comparative functional analyses across both in vitro and in vivo experimental systems demonstrate that CRPC-CAFs confer more robust support for organoid formation and tumor growth relative to CAFs isolated from androgen-dependent prostate tumors. This functional advantage reflects an enhanced capacity of CRPC-CAFs to promote cancer stem cell self-renewal and tumorigenicity 106, 107.

Inactivation of androgen receptor (AR) signaling in prostate CAFs can upregulate LIM domain only 2 (LMO2) expression. LMO2, via paracrine secretion of IL-11 and FGF-9, activates downstream STAT3 and AKT signaling pathways in prostate cancer cells, leading to AR reactivation and ultimately driving castration resistance. Experimental evidence shows that neutralizing antibodies against these cytokines or pathway inhibitors can effectively block this AR reactivation process 108. Additionally, the CD105-positive CAF subpopulation has been reported to promote neuroendocrine differentiation and further induce therapy resistance in prostate tumors via paracrine mechanisms 57. For example, CAFs can confer resistance to anti-androgen therapy by secreting neuregulin 1 (NRG1) to activate the HER3 pathway, and by secreting glucosamine to promote Elk1-mediated transcription of *HSD3B1* 109, 110. Corresponding pharmacological intervention experiments demonstrated that inhibiting the NRG1-HER3 pathway with an NRG1-neutralizing antibody (YW538.24.71) or a HER2/HER3 kinase inhibitor (AMG888) significantly suppressed tumor growth in the 22RV1 xenograft model and restored sensitivity to androgen-targeted therapy 109, 111. AMG888 (patritumab) was evaluated in a Phase I clinical study that included patients with advanced prostate cancer; however, further development was discontinued in 2012. Several lines of evidence have now established NRG1-CAF-HER3 signaling as a key driver of castration resistance in prostate cancer 109, 111; on this basis, patritumab and its ADC derivatives warrant clinical redevelopment. Elk1 inhibition curbs CAF-driven androgen biosynthesis 110. As for CD105⁺ CAFs, a Phase II trial is ongoing that tests carotuximab (ENV-105), a CD105 antagonist, together with apalutamide in metastatic castration-resistant prostate cancer (mCRPC) — early safety and efficacy data appear encouraging.

### Novel Immunomodulatory Therapies Targeting CAFs

Cancer immunomodulation has moved in several new directions, looking well past the immune cells themselves to the TME they live in. In prostate cancer, CAFs orchestrate much of the local immunosuppression, and a growing line of therapy tries to dismantle the physical and immune barriers these fibroblasts build — using natural compounds and novel drugs. Han et al. worked with a polysaccharide (DIP) from the edible fungus *Dictyophora indusiata* and found that it targets the IL-6 secretion pathway in CAFs. This action lifts the brake CAFs put on T cells and improves the immunosuppressive TME 112. Curcumin, for its part, curbs CAF proliferation and selectively clears these cells. It drives a buildup of reactive oxygen species (ROS), setting off endoplasmic reticulum stress and mitochondria-dependent apoptosis — a mechanism that supports combining it with other therapies 113. A measles vaccine strain was turned into an oncolytic virus that makes and secretes a bispecific T-cell engager (BiTE) against FAP and CD3. FAP-positive cancer-associated fibroblasts at sites of viral infection are eliminated by T cells recruited by the measles virus encoding BiTE (MV-BiTE), which also exerts direct tumoricidal effects on malignant cells. Durable anti-tumor immunity is established through the concurrent targeting of both tumor cells and immunosuppressive stromal compartments by this viral agent 114. The dense stroma laid down by CAFs blocks drug entry; nanotechnology-based targeted delivery offers a way around this. Hou et al. constructed a nanoparticle that responds to matrix metalloproteinase-2 (MMP-2), a protease CAFs secrete in abundance. At the tumor site, MMP-2 clips the particles from large to ultra-small, letting them slip through the dense stroma and deliver chemotherapeutics deep into the tumor 115. In a related effort, they guided a nanosystem with an FAP antibody, loaded it with the CXCR4 antagonist AMD3100, and directed it straight to CAFs. Once freed, AMD3100 blocks CXCL12/CXCR4 signaling to pull CAFs back toward a healthier state—giving this approach two layers of targeting 116.

**Figure 3:**
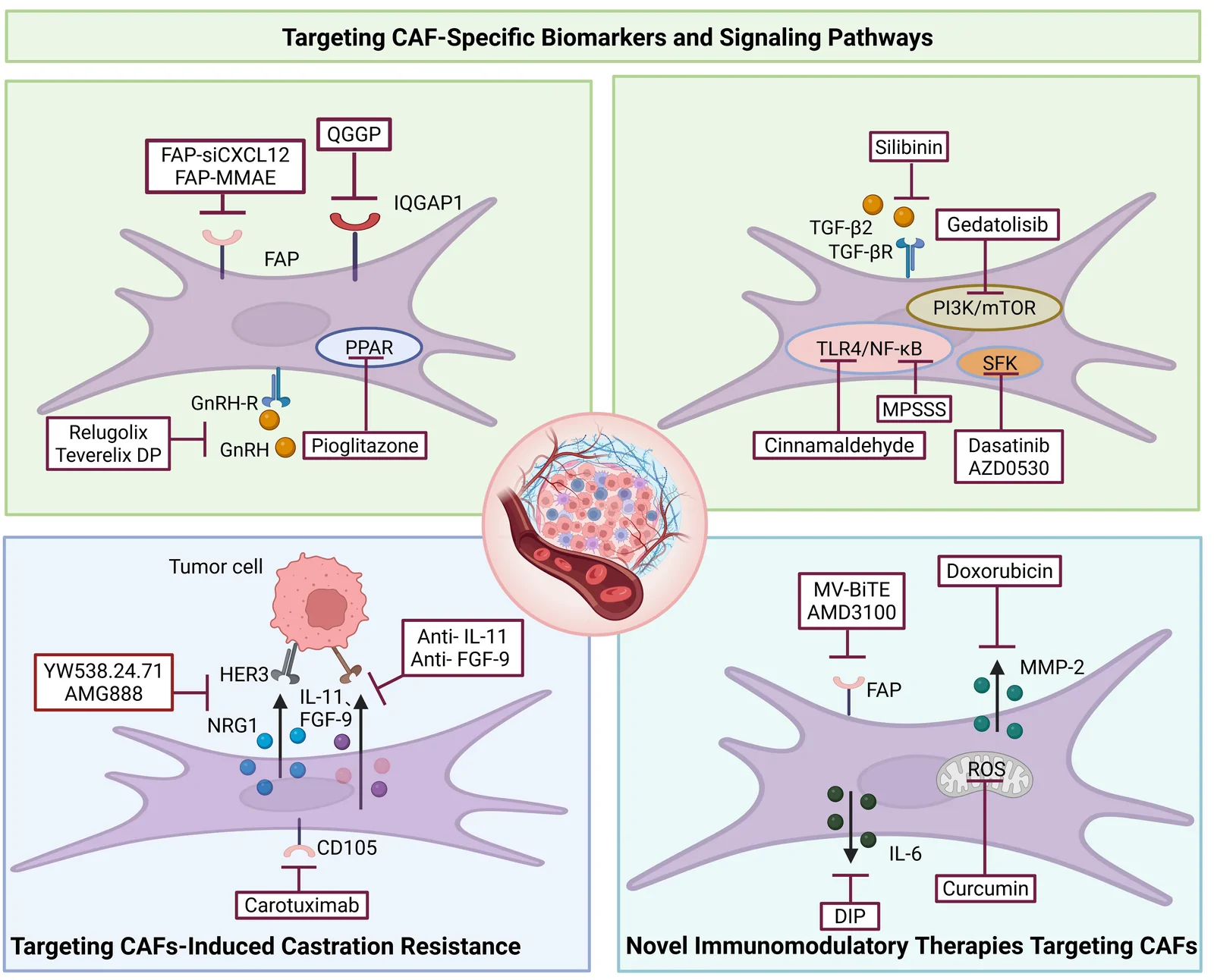
** Several therapeutic strategies are being explored to target CAFs.** CAFs can be targeted via their surface markers (e.g., FAP) and downstream pathways, which blunts their activation and function. Targeting the CAFs that drive castration resistance helps restore sensitivity to androgen therapy. In parallel, natural compounds, oncolytic viruses, and antibody-guided nanomedicines are being developed as immunomodulatory tools, opening new options for treating prostate cancer.

Taken together, prostate cancer CAFs are highly heterogeneous and carry distinct biological traits. Different CAF subsets work through separate molecular routes, and together they set up an immunosuppressive network that drives resistance to immunotherapy. Because of this, hitting CAFs and the pathways they control has become a central approach in combination immunotherapy (Figure 3).

## Summary and Perspectives

CAFs make up the bulk of the stromal compartment in the prostate cancer TME. They were once regarded simply as structural support, but are now seen as active hubs that drive tumor growth, immune escape, and resistance to therapy. This review covers the considerable heterogeneity of CAFs and their plasticity during the shift from hormone-sensitive prostate cancer to CRPC and NEPC. We also examine how CAFs sculpt an immunosuppressive milieu through several routes — directly suppressing CD8⁺ T cells, shaping macrophage polarization, and promoting Treg infiltration. The conversion of the immunologically "cold" prostate cancer TME into an inflamed, immunotherapy-responsive "hot" phenotype can be effectively achieved via functional state reprogramming of defined CAF subsets across disease progression. Complete ablation of immunosuppressive CAF populations represents a central aim of multiple investigational strategies. FAP-directed antibody-drug conjugates deliver microtubule-targeting cytotoxic agents that trigger stromal cell death. Targeted YAP1 blockade drives pro-tumorigenic CAFs toward an immune-supportive phenotype in prostate cancer lesions, which enhances intratumoral CD8⁺ T cell recruitment and elicits anti-tumor immune responses. This intervention represents one strategy to reprogram tumor-promoting CAFs into an anti-tumor functional state. Blockade of the CXCL12-CXCR4 axis mitigates stromal immune exclusion and facilitates T cell infiltration into tumor parenchyma. Disruption of CAF-immune cell crosstalk underlies this distinct therapeutic approach. Such stromal-directed interventions reshape the tumor microenvironment, convert immunologically cold prostate cancer to an inflamed phenotype, and restore sensitivity to immune checkpoint inhibitors. Precise stromal intervention is substantially hindered by this inherent CAF heterogeneity. The full functional spectrum of CAFs remains uncaptured by static classification frameworks that rely on only a limited panel of surface markers. Conflicting prognostic links for individual stromal markers stem from such incomplete characterization, and these discrepancies create a persistent translational challenge.

Spatially resolved crosstalk networks among CAF subpopulations within native three-dimensional tissue architecture have been deciphered with enhanced precision via combined scRNA-seq and spatial transcriptomic approaches in recent investigations. Multi-modal profiling data of this kind validate the pronounced context dependency of CAF functional states, and support a necessary transition in investigative focus away from non-specific stromal depletion toward integrated spatio-temporal analysis. This evolving research strategy incorporates longitudinal sample tracking to map the temporal dynamics of CAF subpopulations throughout disease progression, and pinpoints core regulatory nodes and their associated signaling pathways that drive malignant transformation and immune evasion. Stromal heterogeneity—long regarded as a persistent obstacle to mechanistic stromal biology research—can be reframed as a source of actionable therapeutic targets.

Growing insight into CAF heterogeneity is shifting stroma-directed therapeutic development toward subtype- and functional state-specific modulation for clinical translation. Multi-omics data mining enables identification of druggable targets, including subtype-specific markers such as YAP, TGF-β, and MAOA, as well as signaling pathways enriched in defined pathological stages (e.g., CRPC) or spatially restricted immunosuppressive microenvironments. Intelligent delivery systems, represented by nanotechnological platforms and oncolytic viral vectors, achieve selective targeting of deep tumor regions and individual CAF subtypes. Combination regimens pairing CAF modulators with immune checkpoint inhibitors (ICIs), chemotherapy, or novel hormonal therapies work synergistically to overcome immune tolerance via dual mechanisms: disruption of the stromal physical barrier and alleviation of immunosuppressive signaling. Multidimensional biomarker panels integrating CAF subtype characteristics, spatial distribution profiles, and immune cell functional states enable prediction of immunotherapy response, treatment resistance risk, and overall patient prognosis, and further advance personalized clinical decision-making.

An integrative multi-dimensional stratification framework that unifies functional attributes, spatial localization, and temporal dynamics now governs investigations of prostate cancer CAF heterogeneity, and it has superseded earlier strategies reliant on isolated marker-based annotation. Comprehensive delineation of CAF heterogeneity, phenotypic plasticity, and dynamic reciprocal crosstalk networks with immune cell subsets will require convergent deployment of single-cell and spatial multi-omic platforms, which will be underpinned by state-of-the-art machine learning and artificial intelligence-driven analytical frameworks. Persistent therapeutic bottlenecks that confront current prostate cancer immunotherapy can be effectively addressed via the robust scientific rationale and well-defined translational roadmap derived from this body of work. Mechanistic understanding of immune evasion pathways at the stromal-immune interface in prostate cancer is substantially refined and expanded alongside these research advances. A novel paradigm of personalized, multimodal precision combination immunotherapy focused on tumor microenvironment modulation is poised for development on the basis of these findings.

## Figures and Tables

**Table 1:** CAF subpopulations in prostate cancer

Subtype	Marker Genes	Primary Function	Enriched Region	Clinical/ Prognostic Association	Refs.
myCAFs	αSMA, *Tagln*, *Myl9*	Synthesis and remodeling of the ECM	Peritumoral regions adjacent to cancer cells	-	34
iCAFs	PDGFRα, DPP4, Ly6C	Establishment of an immunosuppressive microenvironment	Hypoxic or perivascular regions	-	34
apCAFs	HLA-DRA, CD74	Antigen presentation	Periphery of TLS	CD74^high^HLA-DRA^high^ signature may predict response to immunotherapy	46
APP-CAFs	CTSK, MRC2	Immunomodulation	-	-	52
myCAFs/ECM-CAFs	α-SMA; POSTN	Excessive ECM deposition, forming a physical barrier	-	-	54
CAFs-C1	*FN1*, *FAP*	ECM remodeling, driving disease progression and resistance to ICIs	CRPC samples	High expression is associated with poor prognosis and ICI resistance	56
CD105⁺ CAFs	CD105	Induction of neuroendocrine differentiation; pro-fibrotic and immunosuppressive myeloid cell recruitment	Surrounding neuroendocrine-differentiated epithelial cells	-	57
HighLM-CAFs	-	Establishment of an immunosuppressive microenvironment	-	Associated with poor response to immunotherapy	58
CAFs-C0	αSMA, CAV1	-	-	-	56
CAF-C1	CTSK, MRC2	Antigen presentation	-	-	52

myCAFs: myofibroblastic CAFs; iCAFs: inflammatory CAFs; apCAFs: antigen-presenting CAFs; APP-CAFs: immune-regulatory/inflammatory CAFs; HighLM-CAFs: high-lactate-metabolism-associated CAFs; ECM: extracellular matrix; CRPC: castration-resistant prostate cancer

**Table 2:** Immune cell regulation by CAFs in prostate cancer: an overview

Subtype	Driver	Target Immune Cell	Mechanism	Biological Effect	Refs.
ECM-CAFs	TGF-β	CD8⁺ T cells	YAP1 binds to IKKα, locking the pro-tumor phenotype; promotes collagen deposition	Impairs CD8⁺ T cell infiltration	66
Lym-CAFs	TNF-α/ IFN-γ	CD8⁺ T cells	High expression of TSG6/CXCL9-11	Recruits and activates CD8⁺ T cells	66
myCAFs	MAOA decline	CD8⁺ T cells	Promotes WNT5A secretion, activating the Ca²⁺-NFATC1 signaling pathway	Increases CD8⁺ T cell infiltration and IFN-γ levels	67
CAFs	TME	CD8⁺ T cells	Secretion of exosomes enriched with PIK3AP1	Inhibits CD8⁺ T cell proliferation and promotes apoptosis	72
CAFs	TME	CD8⁺ T cells	CCL5-CCR5 axis upregulates PD-L1 expression on tumor cells	Suppresses CD8⁺ T cell proliferation and activation	75
CAFs	FOXF2 increase	CD8⁺ T cells, MDSCs	Inhibits CXCL5 promoter activity and the NF-κB-STAT3 pathway, reducing *CXCL5* expression	Increases intratumoral CD8⁺ T cell proportion and function; reduces MDSCs	76
CAFs	TME	Macrophages	Secretion of IL-6, M-CSF, and TGF-β	Promotes monocyte polarization toward M2-type macrophages	81
GPR30^high^ CAFs	GPR30	Macrophages	Upregulates secretion of CXCL12 and IL-6	Recruits macrophages and induces their M2 polarization	82
ADAM12⁺ CAFs	TGF-β	Macrophages, CD8⁺ T cells	High secretion of Gas6, activating the STAT1/3-SOCS axis, reprogramming macrophages, and downregulating CXCL9/10	Drives macrophage M2 polarization; blocks CD8⁺ T cell migration and adhesion	53
CAFs	High glycolysis	Th1, Tregs	Lactate production degrades T-bet via SIRT1 and activates the NF-κB pathway	Inhibits Th1 cell differentiation; promotes Treg cell generation	86
FerroCAFs	Iron	MDSCs	Iron accumulation activates Kdm6b, inducing secretion of CCL2, CSF1, and CXCL1	Recruits MDSCs	87
CAFs. ERα(+)	ERα	Macrophages	Suppresses secretion of chemokines CCL5 and IL6	Reduces macrophage infiltration in the TME	88

ECM-CAFs: ECM-associated CAFs; Lym-CAFs: lymphocyte-associated CAFs; myCAFs: myofibroblastic CAFs; MAOA: monoamine oxidase A; Tregs: regulatory T cells; MDSCs: myeloid-derived suppressor cells; TME: tumor microenvironment; FerroCAFs: iron-laden tumor-associated fibroblasts; ERα: estrogen receptor alpha

**Table 3:** Overview of therapeutic strategies targeting CAFs or related pathways in prostate cancer

Category	Target	Drug	Class	Mechanism	Stage	Refs.
1. Targeting CAF-specific biomarkers & signaling pathways	FAP⁺ CAFs	FAP-siCXCL12	Ab-targeted nanomedicine	Binds CAFs and silences *CXCL12*	Preclinical	92
	FAP⁺ CAFs	FAP-ADC (MMAE)	Antibody-drug conjugate	Targets FAP⁺ CAFs and releases MMAE	Preclinical	93
	CD105⁺ CAFs	Carotuximab	Monoclonal antibody	Blocks CD105-Smad signaling	Phase II	—
	TGF-β2	Silibinin	Natural compound	Inhibits TGF-β2 and CAF transformation	Preclinical	99
	SFK	Dasatinib	Small-molecule inhibitor	Inhibits Src family kinases	Preclinical	95
	SFK	AZD0530	Small-molecule inhibitor	Inhibits Src family kinases	Phase II	96
	TLR4-NF-κB	MPSSS	Natural compound	Activates TLR4-NF-κB to reverse CAF immunosuppression	Preclinical	97
	TLR4-NF-κB	Cinnamaldehyde	Natural compound	Antagonizes TLR4/NF-κB to restore T-cell function	Preclinical	98
	IQGAP1	QGGP	Small-molecule inhibitor	Inhibits IQGAP1 axis to enhance chemosensitivity	Preclinical	19
	PPAR	Pioglitazone	Small-molecule agonist	Reduces PPARγ in PCa cells	Early clinical	101
	GnRH	Relugolix	Small-molecule inhibitor	Suppresses pituitary-gonadal axis	Approved	103
	GnRH	Teverelix DP	Small-molecule inhibitor	Suppresses pituitary-gonadal axis	Phase II	104
	PI3K/mTOR	Gedatolisib	Small-molecule inhibitor	Blocks PI3K/mTOR signaling	Phase II	—
2. Targeting CAF-induced castration resistance	AR(-) CAFs	Anti-IL-11 / Anti-FGF-9 Antibodies	Monoclonal antibodies	Neutralizes IL-11/FGF-9 to block AR reactivation	Preclinical	108
	NRG1-HER3	YW538.24.71	Monoclonal antibody	Blocks NRG1-HER3 signaling	Preclinical	109
	NRG1-HER3	AMG888	Monoclonal antibody	Blocks NRG1-HER3 signaling	Phase I	111
3. Novel immunomodulatory therapies targeting CAFs	FAP⁺ CAFs	MV-BiTE virus	Oncolytic virus	Kills FAP⁺ CAFs and lyses tumor cells	Preclinical	114
	FAP⁺ CAFs	AMD3100	Ab-targeted nanomedicine	Blocks CXCL12/CXCR4 axis to overcome resistance	Preclinical	116
	IL-6	DIP	Natural compound	Inhibits CAF IL-6 secretion to relieve T-cell suppression	Preclinical	112
	CAFs	Curcumin	Natural compound	Inhibits CAF proliferation and viability	Preclinical	113
	CAFs	Doxorubicin	Responsive nanomedicine	MMP-2-cleavable nanoparticle for stromal drug release	Preclinical	115

FAP-ADC: FAP-targeting antibody-drug conjugate; MMAE: monomethyl auristatin E; SFKs: Src family kinases; MPSSS: natural polysaccharide from Lentinula edodes; IQGAP1: IQ motif-containing GTPase-activating protein 1; QGGP: quercetin-3-O-β-D-glucose-7-O-β-D-gentiobioside; PPAR: peroxisome proliferator-activated receptor; GnRH: gonadotropin-releasing hormone; HER3: human epidermal growth factor receptor 3; DIP: polysaccharide from Dictyophora indusiata; BiTE: bispecific T-cell engager; MV-BiTE: measles virus encoding BiTE; MMP-2: matrix metalloproteinase-2

## Abbreviations

ICIs: immune checkpoint inhibitors
PCa: prostate cancer
TME: tumor microenvironment
CAFs: cancer-associated fibroblasts
CAR: chimeric antigen receptor
mCRPC: metastatic castration-resistant prostate cancer
PD-1: programmed cell death protein 1
PSA50: prostate-specific antigen 50% response rate
TAMs: tumor-associated macrophages
myCAFs: myofibroblastic CAFs
iCAFs: inflammatory CAFs
apCAFs: antigen-presenting CAFs
ECM: extracellular matrix
PDAC: pancreatic ductal adenocarcinoma
CRC: colorectal cancer
HGF: hepatocyte growth factor
MHC: major histocompatibility complex
TLS: tertiary lymphoid structures
NSCLC: non-small cell lung cancer
MPR: major pathological response
HSPC: hormone-sensitive prostate cancer
CRPC: castration-resistant prostate cancer
ADT: androgen deprivation therapy
NEPC: neuroendocrine prostate cancer
APP-CAFs: immune-regulatory/inflammatory CAFs
HighLM-CAFs: high-lactate-metabolism-associated CAFs
tCAF: total CAF score
ECM-CAFs: ECM-associated CAFs
Lym-CAFs: lymphocyte-associated CAFs
MAOA: monoamine oxidase A
AKT: protein kinase B
TRAMP: transgenic adenocarcinoma of the mouse prostate
M-CSF: macrophage colony-stimulating factor
EMT: epithelial-mesenchymal transition
SOCS: suppressor of cytokine signaling
FerroCAFs: iron-laden tumor-associated fibroblasts
ERα: estrogen receptor alpha
FAP-ADC: FAP-targeting antibody-drug conjugate
MMAE: monomethyl auristatin E
SFKs: Src family kinases
MPSSS: natural polysaccharide from Lentinula edodes
IQGAP1: IQ motif-containing GTPase-activating protein 1
QGGP: quercetin-3-O-β-D-glucose-7-O-β-D-gentiobioside
OXPHOS: oxidative phosphorylation
ICB: immune checkpoint blockade
PPAR: peroxisome proliferator-activated receptor
GnRH: gonadotropin-releasing hormone
LMO2: LIM domain only 2
NRG1: neuregulin 1
HER3: human epidermal growth factor receptor 3
DIP: polysaccharide from Dictyophora indusiata
BiTE: bispecific T-cell engager
MV-BiTE: measles virus encoding BiTE
MMP-2: matrix metalloproteinase-2

## References

[1] James ND, Tannock I, N'Dow J, Feng F, Gillessen S, Ali SA (2024). The Lancet Commission on prostate cancer: planning for the surge in cases. Lancet.

[2] VanderWeele DJ, Hussain M (2025). Management Decisions for Metastatic Castration-resistant Prostate Cancer in 2024. European Urology.

[3] Van As N, Tree AC, Ostler PJ, van der Voet H, Ford D, Tolan SP (2023). PACE-A: An international phase 3 randomised controlled trial (RCT) comparing stereotactic body radiotherapy (SBRT) to surgery for localised prostate cancer (LPCa)-Primary endpoint analysis. Journal of Clinical Oncology.

[4] Di Bello F, Scheipner L, Baudo A, de Angelis M, Jannello LMI, Siech C (2025). Cancer-specific mortality after radical prostatectomy versus radiotherapy in incidental prostate cancer. Urol Oncol.

[5] Venturi F, Veronesi G, Scotti B, Dika E (2024). Cutaneous Toxicities of Advanced Treatment for Cutaneous Melanoma: A Prospective Study from a Single-Center Institution. Cancers (Basel).

[6] Handel EE, McKeown J, Wei J, Kankaria RA, Burnette H, Johnson DB (2025). Outcomes following long-term disease control with immune checkpoint inhibitors in patients with advanced melanoma. Eur J Cancer.

[7] Anagnostou V, Forde PM, White JR, Niknafs N, Hruban C, Naidoo J (2019). Dynamics of Tumor and Immune Responses during Immune Checkpoint Blockade in Non-Small Cell Lung Cancer. Cancer Res.

[8] Ai X, Jia B, He Z, Zhang J, Zhuo M, Zhao J (2024). Noninvasive early identification of durable clinical benefit from immune checkpoint inhibition: a prospective multicenter study (NCT04566432). Signal Transduct Target Ther.

[9] Labanieh L, Mackall CL (2023). CAR immune cells: design principles, resistance and the next generation. Nature.

[10] Ge Q, Zhao Z, Li X, Yang F, Zhang M, Hao Z (2024). Deciphering the suppressive immune microenvironment of prostate cancer based on CD4+ regulatory T cells: Implications for prognosis and therapy prediction. Clin Transl Med.

[11] Calì B, Troiani M, Bressan S, Attanasio G, Merler S, Moscarda V (2024). Coagulation factor X promotes resistance to androgen-deprivation therapy in prostate cancer. Cancer Cell.

[12] Yu G, Corn PG, Mak CSL, Liang X, Zhang M, Troncoso P (2024). Prostate cancer-induced endothelial-cell-to-osteoblast transition drives immunosuppression in the bone-tumor microenvironment through Wnt pathway-induced M2 macrophage polarization. Proc Natl Acad Sci U S A.

[13] Shackleton EG, Ali HY, Khan M, Pockley GA, McArdle SE (2021). Novel Combinatorial Approaches to Tackle the Immunosuppressive Microenvironment of Prostate Cancer. Cancers (Basel).

[14] Novysedlak R, Guney M, Al Khouri M, Bartolini R, Koumbas Foley L, Benesova I (2025). The Immune Microenvironment in Prostate Cancer: A Comprehensive Review. Oncology.

[15] Petrylak DP, Ratta R, Matsubara N, Korbenfeld E, Gafanov R, Mourey L (2025). Pembrolizumab Plus Docetaxel Versus Docetaxel for Previously Treated Metastatic Castration-Resistant Prostate Cancer: The Randomized, Double-Blind, Phase III KEYNOTE-921 Trial. J Clin Oncol.

[16] Nguyen CB, Reimers MA, Perera C, Abida W, Chou J, Feng FY (2024). Evaluating Immune Checkpoint Blockade in Metastatic Castration-Resistant Prostate Cancers with Deleterious CDK12 Alterations in the Phase 2 IMPACT Trial. Clin Cancer Res.

[17] Syed A, Raza H, Khaskheli HK, Rafique I, Shahid S, Shahzadi N (2025). Assessing the clinical outcomes of immunotherapy and docetaxel combinations in metastatic castration-resistant prostate cancer: a meta-analysis. BMC Cancer.

[18] Lai C, Wu Z, Li Z, Huang X, Hu Z, Yu H (2024). Single-cell analysis extracted CAFs-related genes to established online app to predict clinical outcome and radiotherapy prognosis of prostate cancer. Clin Transl Oncol.

[19] Xiong Z, Zhuang R-L, Yu S-L, Xie Z-X, Peng S-R, Li Z-A (2025). Cancer-associated fibroblasts regulate mitochondrial metabolism and inhibit chemosensitivity via ANGPTL4-IQGAP1 axis in prostate cancer. Journal of Advanced Research.

[20] Cheng B, Yu Q, Wang W (2023). Intimate communications within the tumor microenvironment: stromal factors function as an orchestra. J Biomed Sci.

[21] Spary LK, Salimu J, Webber JP, Clayton A, Mason MD, Tabi Z (2014). Tumor stroma-derived factors skew monocyte to dendritic cell differentiation toward a suppressive CD14(+) PD-L1(+) phenotype in prostate cancer. Oncoimmunology.

[22] Mao X, Xu J, Wang W, Liang C, Hua J, Liu J (2021). Crosstalk between cancer-associated fibroblasts and immune cells in the tumor microenvironment: new findings and future perspectives. Mol Cancer.

[23] Kwon WA, Joung JY (2025). Immunotherapy in Prostate Cancer: From a "Cold" Tumor to a "Hot" Prospect. Cancers (Basel).

[24] Cords L, Tietscher S, Anzeneder T, Langwieder C, Rees M, de Souza N (2023). Cancer-associated fibroblast classification in single-cell and spatial proteomics data. Nat Commun.

[25] Lin JR, Chen YA, Campton D, Cooper J, Coy S, Yapp C (2023). High-plex immunofluorescence imaging and traditional histology of the same tissue section for discovering image-based biomarkers. Nat Cancer.

[26] Huang Z, Chen J, Zhu T, Li J, Ng HY, Zhou Y (2025). Cancer-associated fibroblasts in the tumor microenvironment: heterogeneity, crosstalk mechanisms, and therapeutic implications. Mol Cancer.

[27] Liu L, Wan W, Chang Y, Ao L, Xu Y, Xu X (2025). Crosstalk between heterogeneous cancer-associated fibroblast subpopulations and the immune system in breast cancer: key players and promising therapeutic targets. J Exp Clin Cancer Res.

[28] Lin Z, Li G, Jiang K, Li Z, Liu T (2024). Cancer therapy resistance mediated by cancer-associated fibroblast-derived extracellular vesicles: biological mechanisms to clinical significance and implications. Mol Cancer.

[29] Flynn JM, Thadani N, Gallagher EE, Azzaro I, Bodnar CM, McCarty CP (2025). Plasticity and Functional Heterogeneity of Cancer-Associated Fibroblasts. Cancer Res.

[30] Rantanen F, Murumägi A, Arjama M, Välimäki K, Multamäki E, Mirtti T (2025). Molecular profiling of ex vivo prostate cancer CAF models captures stromal heterogeneity and drug vulnerabilities. Cell Death Discov.

[31] Liu Y, Sinjab A, Min J, Han G, Paradiso F, Zhang Y (2025). Conserved spatial subtypes and cellular neighborhoods of cancer-associated fibroblasts revealed by single-cell spatial multi-omics. Cancer Cell.

[32] Raaijmakers K, Adema GJ, Bussink J, Ansems M (2024). Cancer-associated fibroblasts, tumor and radiotherapy: interactions in the tumor micro-environment. J Exp Clin Cancer Res.

[33] Cantallops Vilà P, Ravichandra A, Agirre Lizaso A, Perugorria MJ, Affò S (2024). Heterogeneity, crosstalk, and targeting of cancer-associated fibroblasts in cholangiocarcinoma. Hepatology.

[34] Öhlund D, Handly-Santana A, Biffi G, Elyada E, Almeida AS, Ponz-Sarvise M (2017). Distinct populations of inflammatory fibroblasts and myofibroblasts in pancreatic cancer. J Exp Med.

[35] Sun Y, Qiao Y, Niu Y, Madhavan BK, Fang C, Hu J (2025). ARP2/3 complex affects myofibroblast differentiation and migration in pancreatic ductal adenocarcinoma. Int J Cancer.

[36] Pang N, Yang Z, Zhang W, Du Y, Zhang L, Li X (2024). Cancer-associated fibroblasts barrier breaking via TGF-β blockade paved way for docetaxel micelles delivery to treat pancreatic cancer. International Journal of Pharmaceutics.

[37] Miao C, Liu L, Cao Y, Jiang Z, Ding Z, Chen Y (2024). OSCC-derived EVs educate fibroblasts and remodel collagen landscape. Matrix Biology.

[38] You R, Shen Q, Lin C, Dong K, Liu X, Xu H (2025). Single-cell and spatial transcriptomics reveal mechanisms of radioresistance and immune escape in recurrent nasopharyngeal carcinoma. Nat Genet.

[39] Chen B, Chan WN, Xie F, Mui CW, Liu X, Cheung AHK (2023). The molecular classification of cancer-associated fibroblasts on a pan-cancer single-cell transcriptional atlas. Clin Transl Med.

[40] Menezes S, Okail MH, Jalil SMA, Kocher HM, Cameron AJM (2022). Cancer-associated fibroblasts in pancreatic cancer: new subtypes, new markers, new targets. J Pathol.

[41] Mosa MH, Michels BE, Menche C, Nicolas AM, Darvishi T, Greten FR (2020). A Wnt-Induced Phenotypic Switch in Cancer-Associated Fibroblasts Inhibits EMT in Colorectal Cancer. Cancer Res.

[42] Wang H, Li N, Liu Q, Guo J, Pan Q, Cheng B (2023). Antiandrogen treatment induces stromal cell reprogramming to promote castration resistance in prostate cancer. Cancer Cell.

[43] Biffi G, Oni TE, Spielman B, Hao Y, Elyada E, Park Y (2019). IL1-Induced JAK/STAT Signaling Is Antagonized by TGFβ to Shape CAF Heterogeneity in Pancreatic Ductal Adenocarcinoma. Cancer Discov.

[44] Chen X, Zhou Z, Xie L, Qiao K, Jia Y, Liu S (2025). Single-cell resolution spatial analysis of antigen-presenting cancer-associated fibroblast niches. Cancer Cell.

[45] Elyada E, Bolisetty M, Laise P, Flynn WF, Courtois ET, Burkhart RA (2019). Cross-Species Single-Cell Analysis of Pancreatic Ductal Adenocarcinoma Reveals Antigen-Presenting Cancer-Associated Fibroblasts. Cancer Discov.

[46] Cao Z, Meng Z, Li J, Tian Y, Lu L, Wang A (2025). Interferon-γ-stimulated antigen-presenting cancer-associated fibroblasts hinder neoadjuvant chemoimmunotherapy efficacy in lung cancer. Cell Rep Med.

[47] Song J, Wei R, Liu C, Zhao Z, Liu X, Wang Y (2025). Antigen-presenting cancer associated fibroblasts enhance antitumor immunity and predict immunotherapy response. Nat Commun.

[48] Cui X, Liu S, Song H, Xu J, Sun Y (2025). Single-cell and spatial transcriptomic analyses revealing tumor microenvironment remodeling after neoadjuvant chemoimmunotherapy in non-small cell lung cancer. Mol Cancer.

[49] Zhang N, Harbers L, Simonetti M, Diekmann C, Verron Q, Berrino E (2024). High clonal diversity and spatial genetic admixture in early prostate cancer and surrounding normal tissue. Nat Commun.

[50] Davies AH, Beltran H, Zoubeidi A (2018). Cellular plasticity and the neuroendocrine phenotype in prostate cancer. Nat Rev Urol.

[51] Aggarwal R, Huang J, Alumkal JJ, Zhang L, Feng FY, Thomas GV (2018). Clinical and Genomic Characterization of Treatment-Emergent Small-Cell Neuroendocrine Prostate Cancer: A Multi-institutional Prospective Study. J Clin Oncol.

[52] Wang W, Li T, Xie Z, Zhao J, Zhang Y, Ruan Y (2024). Integrating single-cell and bulk RNA sequencing data unveils antigen presentation and process-related CAFS and establishes a predictive signature in prostate cancer. J Transl Med.

[53] Di Carlo SE, Raffenne J, Varet H, Ode A, Granados DC, Stein M (2023). Depletion of slow-cycling PDGFRα(+)ADAM12(+) mesenchymal cells promotes antitumor immunity by restricting macrophage efferocytosis. Nat Immunol.

[54] Masuda H (2025). Cancer-associated fibroblasts in cancer drug resistance and cancer progression: a review. Cell Death Discov.

[55] Cheng Y, Liu B, Xin J, Wu X, Li W, Shang J (2025). Single-cell and spatial RNA sequencing identify divergent microenvironments and progression signatures in early- versus late-onset prostate cancer. Nat Aging.

[56] Pan J, Ma Z, Liu B, Qian H, Shao X, Liu J (2023). Identification of cancer-associated fibroblasts subtypes in prostate cancer. Front Immunol.

[57] Kato M, Placencio-Hickok VR, Madhav A, Haldar S, Tripathi M, Billet S (2019). Heterogeneous cancer-associated fibroblast population potentiates neuroendocrine differentiation and castrate resistance in a CD105-dependent manner. Oncogene.

[58] Ji S, Xi Z, Li T, Jia G, Zhang Y, Zheng C (2025). PA2G4 in CAFs promotes biochemical recurrence of prostate cancer via H3K18la. Cell Biosci.

[59] Monteran L, Erez N (2019). The Dark Side of Fibroblasts: Cancer-Associated Fibroblasts as Mediators of Immunosuppression in the Tumor Microenvironment. Front Immunol.

[60] Qiu Y, Wang Y, Liu J, Sun K, Liu B, Hou Q (2025). Single-cell sequencing unveils the transcriptomic landscape of castration-resistant prostate cancer-associated fibroblasts and their association with prognosis and immunotherapy response. BMC Cancer.

[61] Gao Z, Zhang N, An B, Li D, Fang Z, Xu D (2024). Comprehensive analyses of the cancer-associated fibroblast subtypes and their score system for prediction of outcomes and immunosuppressive microenvironment in prostate cancer. Cancer Cell Int.

[62] Wen XY, Wang RY, Yu B, Yang Y, Yang J, Zhang HC (2023). Integrating single-cell and bulk RNA sequencing to predict prognosis and immunotherapy response in prostate cancer. Sci Rep.

[63] Talia M, Cesario E, Cirillo F, Scordamaglia D, Di Dio M, Zicarelli A (2024). Cancer-associated fibroblasts (CAFs) gene signatures predict outcomes in breast and prostate tumor patients. J Transl Med.

[64] Xu W, Liu S, Ma L, Cheng L, Li Q, Qing L (2024). Identification of miRNA signature in cancer-associated fibroblast to predict recurrent prostate cancer. Comput Biol Med.

[65] Ruff M, Leyme A, Le Cann F, Bonnier D, Le Seyec J, Chesnel F (2015). The Disintegrin and Metalloprotease ADAM12 Is Associated with TGF-β-Induced Epithelial to Mesenchymal Transition. PLoS One.

[66] Song H, Lu T, Han D, Zhang J, Gan L, Xu C (2024). YAP1 Inhibition Induces Phenotype Switching of Cancer-Associated Fibroblasts to Tumor Suppressive in Prostate Cancer. Cancer Res.

[67] Zhao Z, Hu Y, Li H, Lu T, He X, Ma Y (2025). Inhibition of stromal MAOA leading activation of WNT5A enhance prostate cancer immunotherapy by involving the transition of cancer-associated fibroblasts. J Immunother Cancer.

[68] Federspiel J, Dudas J, Hofauer BG, Wollenberg B, Steinbichler TB (2025). Cancer-Associated Fibroblasts-Derived Exosomes as Mediators of Immunotherapy Resistance in Head and Neck Squamous Cell Carcinoma. Cells.

[69] Gou Z, Li J, Liu J, Yang N (2024). The hidden messengers: cancer associated fibroblasts-derived exosomal miRNAs as key regulators of cancer malignancy. Front Cell Dev Biol.

[70] Nedaeinia R, Najafgholian S, Salehi R, Goli M, Ranjbar M, Nickho H (2024). The role of cancer-associated fibroblasts and exosomal miRNAs-mediated intercellular communication in the tumor microenvironment and the biology of carcinogenesis: a systematic review. Cell Death Discov.

[71] Wang S, Du P, Cao Y, Ma J, Yang X, Yu Z (2022). Cancer associated fibroblasts secreted exosomal miR-1290 contributes to prostate cancer cell growth and metastasis via targeting GSK3β. Cell Death Discov.

[72] Li T, Yi C, Xiang Z, You X, Yu J (2025). Cancer-associated fibroblasts-derived exosome PIK3AP1 promotes the malignant progression and immune escape of prostate cancer cells. Pathol Res Pract.

[73] Yan R, Moresco P, Gegenhuber B, Fearon DT (2023). T cell-Mediated Development of Stromal Fibroblasts with an Immune-Enhancing Chemokine Profile. Cancer Immunol Res.

[74] Gorchs L, Fernández Moro C, Bankhead P, Kern KP, Sadeak I, Meng Q (2019). Human Pancreatic Carcinoma-Associated Fibroblasts Promote Expression of Co-inhibitory Markers on CD4(+) and CD8(+) T-Cells. Front Immunol.

[75] Xiong Z, Yu SL, Xie ZX, Zhuang RL, Peng SR, Wang Q (2024). Cancer-associated fibroblasts promote enzalutamide resistance and PD-L1 expression in prostate cancer through CCL5-CCR5 paracrine axis. iScience.

[76] Jia D, Zhou Z, Kwon OJ, Zhang L, Wei X, Zhang Y (2022). Stromal FOXF2 suppresses prostate cancer progression and metastasis by enhancing antitumor immunity. Nat Commun.

[77] Chen S, Saeed A, Liu Q, Jiang Q, Xu H, Xiao GG (2023). Macrophages in immunoregulation and therapeutics. Signal Transduct Target Ther.

[78] Gu Q, Qi A, Wang N, Zhou Z, Zhou X (2024). Macrophage dynamics in prostate cancer: Molecular to therapeutic insights. Biomed Pharmacother.

[79] Li W, Chen S, Lu J, Mao W, Zheng S, Zhang M (2025). YY1 enhances HIF-1α stability in tumor-associated macrophages to suppress anti-tumor immunity of prostate cancer in mice. Nat Commun.

[80] Chen S, Lu K, Hou Y, You Z, Shu C, Wei X (2023). YY1 complex in M2 macrophage promotes prostate cancer progression by upregulating IL-6. J Immunother Cancer.

[81] Comito G, Giannoni E, Segura CP, Barcellos-de-Souza P, Raspollini MR, Baroni G (2014). Cancer-associated fibroblasts and M2-polarized macrophages synergize during prostate carcinoma progression. Oncogene.

[82] Zhang R, Zong J, Peng Y, Shi J, Du X, Liu H (2021). GPR30 knockdown weakens the capacity of CAF in promoting prostate cancer cell invasion via reducing macrophage infiltration and M2 polarization. J Cell Biochem.

[83] Sun Y, Ren S, Wen W, Jing J, Luo X, Shao S (2025). Targeting CXCR2 in prostate cancer cells can block CD47-SIRPα interaction and reverse M2 macrophage polarization in the TME. Mol Cancer.

[84] Woods AN, Wilson AL, Srivinisan N, Zeng J, Dutta AB, Peske JD (2017). Differential Expression of Homing Receptor Ligands on Tumor-Associated Vasculature that Control CD8 Effector T-cell Entry. Cancer Immunol Res.

[85] Jia H, Chen X, Zhang L, Chen M (2025). Cancer associated fibroblasts in cancer development and therapy. J Hematol Oncol.

[86] Comito G, Iscaro A, Bacci M, Morandi A, Ippolito L, Parri M (2019). Lactate modulates CD4(+) T-cell polarization and induces an immunosuppressive environment, which sustains prostate carcinoma progression via TLR8/miR21 axis. Oncogene.

[87] Zhang K, Liu K, Hu B, Du G, Chen X, Xiao L (2024). Iron-loaded cancer-associated fibroblasts induce immunosuppression in prostate cancer. Nat Commun.

[88] Yeh CR, Slavin S, Da J, Hsu I, Luo J, Xiao GQ (2016). Estrogen receptor α in cancer associated fibroblasts suppresses prostate cancer invasion via reducing CCL5, IL6 and macrophage infiltration in the tumor microenvironment. Mol Cancer.

[89] Yu H, Wang Y, He M, Chen Y, Bi J, Lin X (2026). Integrated single-cell and spatial transcriptomic profiling decodes lineage plasticity and immune microenvironment remodeling in prostate cancer progression. Mol Cancer.

[90] Kiani M, Jokar S, Hassanzadeh L, Behnammanesh H, Bavi O, Beiki D (2024). Recent Clinical Implications of FAPI: Imaging and Therapy. Clin Nucl Med.

[91] Jiang GM, Xu W, Du J, Zhang KS, Zhang QG, Wang XW (2016). The application of the fibroblast activation protein α-targeted immunotherapy strategy. Oncotarget.

[92] Lang J, Zhao X, Qi Y, Zhang Y, Han X, Ding Y (2019). Reshaping Prostate Tumor Microenvironment To Suppress Metastasis via Cancer-Associated Fibroblast Inactivation with Peptide-Assembly-Based Nanosystem. ACS Nano.

[93] Gallant JP, Hintz HM, Gunaratne GS, Breneman MT, Recchia EE, West JL (2024). Mechanistic Characterization of Cancer-associated Fibroblast Depletion via an Antibody-Drug Conjugate Targeting Fibroblast Activation Protein. Cancer Res Commun.

[94] Kakarla M, ChallaSivaKanaka S, Dufficy MF, Gil V, Filipovich Y, Vickman R (2022). Ephrin B Activate Src Family Kinases in Fibroblasts Inducing Stromal Remodeling in Prostate Cancer. Cancers (Basel).

[95] Park SI, Zhang J, Phillips KA, Araujo JC, Najjar AM, Volgin AY (2008). Targeting SRC family kinases inhibits growth and lymph node metastases of prostate cancer in an orthotopic nude mouse model. Cancer Res.

[96] Lara PN Jr, Longmate J, Evans CP, Quinn DI, Twardowski P, Chatta G (2009). A phase II trial of the Src-kinase inhibitor AZD0530 in patients with advanced castration-resistant prostate cancer: a California Cancer Consortium study. Anticancer Drugs.

[97] Xu Y, Ma J, Zheng Q, Wang Y, Hu M, Ma F (2019). MPSSS impairs the immunosuppressive function of cancer-associated fibroblasts via the TLR4-NF-κB pathway. Biosci Rep.

[98] Mei J, Ma J, Xu Y, Wang Y, Hu M, Ma F (2020). Cinnamaldehyde Treatment of Prostate Cancer-Associated Fibroblasts Prevents Their Inhibitory Effect on T Cells Through Toll-Like Receptor 4. Drug Des Devel Ther.

[99] Ting HJ, Deep G, Jain AK, Cimic A, Sirintrapun J, Romero LM (2015). Silibinin prevents prostate cancer cell-mediated differentiation of naïve fibroblasts into cancer-associated fibroblast phenotype by targeting TGF β2. Mol Carcinog.

[100] Zhai X, Chen X, Wan Z, Ge M, Ding Y, Gu J (2023). Identification of the novel therapeutic targets and biomarkers associated of prostate cancer with cancer-associated fibroblasts (CAFs). Front Oncol.

[101] Suzuki S, Mori Y, Nagano A, Naiki-Ito A, Kato H, Nagayasu Y (2016). Pioglitazone, a Peroxisome Proliferator-Activated Receptor γ Agonist, Suppresses Rat Prostate Carcinogenesis. Int J Mol Sci.

[102] Atas E, Berchtold K, Schlederer M, Prodinger S, Sternberg F, Pucci P (2025). The anti-diabetic PPARγ agonist Pioglitazone inhibits cell proliferation and induces metabolic reprogramming in prostate cancer. Mol Cancer.

[103] Shore ND, Saad F, Cookson MS, George DJ, Saltzstein DR, Tutrone R (2020). Oral Relugolix for Androgen-Deprivation Therapy in Advanced Prostate Cancer. N Engl J Med.

[104] Ulys A, Jankevicus F, Jievaltas M, Venckus R, Auskalnis S, Kardelis Z (2024). Efficacy, tolerability, and safety of teverelix DP in patients with advanced prostate cancer: A multicenter, open-label, phase 2 trial. Prostate.

[105] Pan S, Yin R, Zhu H, Shen S, Li Z, Liu B (2024). Prostate cancer cancer-associated fibroblasts with stable markers post-androgen deprivation therapy associated with tumor progression and castration resistant prostate cancer. Cancer Sci.

[106] Adisetiyo H, Liang M, Liao CP, Jeong JH, Cohen MB, Roy-Burman P (2014). Dependence of castration-resistant prostate cancer (CRPC) stem cells on CRPC-associated fibroblasts. J Cell Physiol.

[107] Chen H, Fang S, Zhu X, Liu H (2024). Cancer-associated fibroblasts and prostate cancer stem cells: crosstalk mechanisms and implications for disease progression. Front Cell Dev Biol.

[108] Chen L, Wang YY, Li D, Wang C, Wang SY, Shao SH (2021). LMO2 upregulation due to AR deactivation in cancer-associated fibroblasts induces non-cell-autonomous growth of prostate cancer after androgen deprivation. Cancer Lett.

[109] Zhang Z, Karthaus WR, Lee YS, Gao VR, Wu C, Russo JW (2020). Tumor Microenvironment-Derived NRG1 Promotes Antiandrogen Resistance in Prostate Cancer. Cancer Cell.

[110] Cui D, Li J, Zhu Z, Berk M, Hardaway A, McManus J (2023). Cancer-associated fibroblast-secreted glucosamine alters the androgen biosynthesis program in prostate cancer via HSD3B1 upregulation. J Clin Invest.

[111] Gil V, Miranda S, Riisnaes R, Gurel B, D'Ambrosio M, Vasciaveo A (2021). HER3 Is an Actionable Target in Advanced Prostate Cancer. Cancer Res.

[112] Han S, Ma C, Hu M, Wang Y, Ma F, Tao N (2017). A polysaccharide from Dictyophora indusiata inhibits the immunosuppressive function of cancer-associated fibroblasts. Cell Biochem Funct.

[113] Zeng Y, Du Q, Zhang Z, Ma J, Han L, Wang Y (2020). Curcumin promotes cancer-associated fibroblasts apoptosis via ROS-mediated endoplasmic reticulum stress. Arch Biochem Biophys.

[114] Freedman JD, Duffy MR, Lei-Rossmann J, Muntzer A, Scott EM, Hagel J (2018). An Oncolytic Virus Expressing a T-cell Engager Simultaneously Targets Cancer and Immunosuppressive Stromal Cells. Cancer Res.

[115] Hou L, Chen D, Hao L, Tian C, Yan Y, Zhu L (2019). Transformable nanoparticles triggered by cancer-associated fibroblasts for improving drug permeability and efficacy in desmoplastic tumors. Nanoscale.

[116] Li J, Lei T, Ouyang W, Ye Z, Li L, Li G (2025). Reshape tumor microenvironment by modulating CXCR4 with FAP-targeted diselenide-organosilica delivery system for prostate cancer immunotherapy. Chemical Engineering Journal.

